# Where on earth to publish? A sample survey comparing traditional and open access publishing in the oncological field

**DOI:** 10.1186/1756-9966-32-4

**Published:** 2013-01-22

**Authors:** Elisabetta Poltronieri, Elena Bravo, Tiziana Camerini, Maurizio Ferri, Roberto Rizzo, Renata Solimini, Gaetana Cognetti

**Affiliations:** 1 Publishing Unit, Istituto Superiore di Sanità, Rome, Italy; 2 Department of Cell Biology and Neurosciences, Istituto Superiore di Sanità, Rome, Italy; 3 Fondazione IRCCS Istituto Nazionale dei Tumori, Milan, Italy; 4 Data Management Unit, Istituto Superiore di Sanità, Rome, Italy; 5 Scientific and Patient Library, Regina Elena National Cancer Institute, Rome, Italy; 6 Department of Therapeutic Research and Medicines Evaluation, Istituto Superiore di Sanità, Rome, Italy

**Keywords:** Open access publishing, Scientific publications, Oncology, Surveys, Italy

## Abstract

**Background:**

The paper intends to help scientific authors to make the best choice of journals in which to publish, by describing and comparing journal features in the area of oncology. For this purpose, the authors identified impact factor (IF) ranking, cost options and copyright conditions offered to authors wishing to publish in full open access (OA), subscription-based or hybrid journals.

**Methods:**

Data referring to articles published in 2010 by three Italian research institutions (National Institute of Health – Rome (ISS), Regina Elena National Cancer Institute – Rome (IRE), National Cancer Institute – Milan (INT) in journals (78) managed according to different business models, all listed in the Journal Citation Reports, subject category Oncology, were collected and analysed. The journals surveyed were ranked according to IF, position in quartiles, publication charges, usage rights in published articles, self-archiving conditions in OAI-compliant repositories digital archives.

**Results:**

Almost half (34) the journals surveyed were included in the first quartile, thus revealing authors’ preference for journals with a high IF. The prevalent journal business model was the hybrid formula (based on subscriptions but also offering a paid OA option) with 51 journals, followed by subscription-based only journals accounting for 22, while just 5 full OA journals were identified. In general, no relationship was found between IF and article publication charges, in terms of correspondence between more expensive fees and higher IF.

**Conclusions:**

The issue of OA journals as compared with traditional subscription-based journals is highly debated among stakeholders: library administrators facing financial restrictions, authors seeking to locate the best outlet for their research, publishers wishing to increase their revenues by offering journals with wider appeal. Against this background, factors such as the quest for alternatives to high-cost business models, investments in setting up institutional repositories hosting the published versions of articles and efforts to overcome copyright barriers and gain free access to scientific literature are all crucial.

## Background

"“If you have knowledge, let others light their candles with it”"

"Winston Churchill"

"“The key question is whether there are new opportunities and new models for scholarly publishing that would better serve researchers and better communicate and disseminate research findings”*"

"*Digital broadband Content: Scientific Publishing, OECD, Paris. Available from: http://www.oecd.org/internet/interneteconomy/35393145.pdf"

Open access (OA) paradigm has unquestionably reshaped the traditional system of scholarly communication by closely linking the concepts of free access to research literature and its easy diffusion and re-use through massive exploitation of Internet and related technologies and an innovative management of copyright rules.

OA journals based on an “author-pays” business model are one of the two routes of the open access paradigm, the so called “gold” route. Golden OA is complementary to “green” OA, founded on depositing accepted manuscripts in institutional or discipline-based repositories.

In offering a comprehensive overview of the OA movement, including its history and achievements, Peter Suber defines OA journals as: peer-reviewed journals available online to the reader "without financial, legal, or technical barriers other than those inseparable from gaining access to the internet itself" [1].

Recent studies of the economic implications of access to journal articles highlight the present and future scenarios of the scholarly publication system [2-5]. The core objective of these studies is to verify the sustainability of the OA paradigm compared with the traditional model, in order to create conditions to achieve savings and increase both efficiency and effectiveness of scientific communication for the scholarly community.

The commercial publishing models and copyright policies of scholarly journals considered in this survey are:

1. Traditional, subscription-based journals that allow access to their articles only upon the payment of a subscription fee. In this case, publishers often require that authors transfer copyright ownership to them as a condition of publication. Therefore, authors are usually required to sign a Copyright Transfer Agreement (CTA) or an Exclusive Licence Form (ELF).

2. Full or pure open-access journals that make their content freely available online. These journals allow authors to retain the copyright of their work and rely on publication fees - so called Article Processing Charges (APC) - paid by the authors, their institutions or funders.

3. Hybrid open-access journals, subscription-based journals offering an OA option to authors, by asking them to pay an additional fee to allow free access to their articles online. In this case, publishers may decide not to allow authors to retain the copyright in their work.

Authors of scientific publications in the biomedical field thus have a wide choice of alternatives, according to whether publishers adhere fully or partially to the OA publishing model. This implies that authors should indeed learn to choose the journal that best fits their needs and expectations, in terms of quality contents, affordable costs, wide impact of research findings and, last but not least, copyright conditions. In brief, authors need to have the knowledge and tools to help them cope with the numerous options offered by publishers of scientific journals. Table S 1 summarises some major factors that authors should consider when deciding which journal best meets their needs.

This study aimed to find the most satisfactory balance between the basic “ingredients” of scientific publishing practices. Some of its findings may also be useful to stakeholders when deciding whether or not to implement OAI-compliant digital/institutional archives and to manage OA journals at their institutions or at a national level in a shared, co-operative way.

## Methods

The survey, carried out in the first semester of 2012, identified collected and analysed journals hosting articles published in 2010 and authored by the medical and research staff of three Italian research institutions: the Istituto Superiore di Sanità, ISS (Department of Haematology, Oncology and Molecular Medicine, Rome); the Istituto Regina Elena, IRE, Rome; and the Fondazione IRCCS Istituto Nazionale Tumori, INT, Milan. Some of the scientists affiliated with IRE and INT work in the experimental and some in the clinical field of oncology, while most ISS authors perform their research in experimental medicine, including oncology.

Data relating to the journal articles were extracted from the institutional archives of the three institutes. The journals considered in the survey were those in the Oncology subject category of the Journal Citation Reports released by Thomson Reuters (JCR, Science Edition 2010) [6]. The journal impact factor (IF) 2010 and quartile (Q) ranking position for each journal were also retrieved from JCR.

Journals are generally sorted into quartiles for research evaluation systems in order to overcome the bias related to the direct comparison of the IF scores of journals that are listed in diverse subject areas. Quartiles, a division into four equal percentiles of the journals listed in a category, are also used by the Italian Ministry of Health to evaluate publications authored by the research institutes of the National Health Service [7,8].

The survey examined publishers, business models (subscription-based, full open access, hybrid open access), and publication fees per article. To allow easy price comparisons, the costs were also calculated in euros where prices were reported only in US dollars and/or GB pounds, according to the exchange rate of 27 August 2012. It should be noted that authors are sometimes charged additional costs for extra pages, colour tables or figures, reprints, etc.

Data relating to the journals’ business models were retrieved by searching the SHERPA/RoMEO [9] database which draws a distinction between the following journal categories: subscription-based journals, full OA and hybrid OA journals. This database was also a privileged source of information for quickly identifying features of the single journals surveyed, such as the publisher’s name and copyright policy, in regard to both the regulation of intellectual property rights and the level of openness of self-archiving. With respect to this latter point, the SHERPA/RoMEO database groups publishers in four different colours, from those with more permissive conditions to those with a stricter approach, as follows: green indicates publishers that permit archiving of pre-print, and post-print or publisher’s version/PDF; blue indicates those that allow archiving of post-print (i.e. final draft post-refereeing) or publisher's version/PDF; and yellow those that permit archiving of pre-print (i.e. pre-refereeing); white indicates publishers that do not support any archiving.

Other aspects considered in this survey concern the copyright policies relating to current publishers of the journals listed in Table S 2. The most widely used models are: Copyright Transfer Agreement (CTA), Exclusive Licence Form (ELF) and Creative Commons Attribution (CCA). The author signing the CTA transfers all exploitation rights (in terms of re-use and redistribution of an article for educational or commercial purposes) to the publisher, except the moral ones (paternity and integrity rights). Within this model, thanks to the constant pressure exerted by the OA movement against all kinds of barrier (legal, economical and technical) to free access to research output, authors now usually have a wide range of innovative choices compared with traditional copyright transfer statements. In fact, some large publishers, such as Elsevier and Wiley-Blackwell, include a clause in their CTAs in which they licence back to authors some non-commercial rights for scholarly or educational purposes (i.e. teaching use, sharing copies among colleagues, making articles freely-accessible online by placing them in institutional repositories). This model thus increasingly resembles the ELF, which leaves the copyright with the author, but assigns to the publisher the exclusive right to publish the work. The ELF has the advantage that the author remains free to use or re-use the work, usually not for direct commercial use, without needing to ask permission.

A third copyright model, proposed by a small percentage of publishers, is that known as CCA, promoted by the Creative Commons Corporation [10], a US non-profit organisation founded in 2001, inspired by the OA paradigm and the open source software movement. More precisely, CC licences [11] guarantee a balance between protection and access by permitting some re-use without the need to ask publishers for specific permission. There are six types of CC licence, ranging from the least restrictive (*attribution*, used by pioneer OA publishers PloS and BioMed Central) to the more restrictive (*attribution*, *non-commercial*, *no derivative works*). The least restrictive model recognises the intellectual property rights of the author, while the most restricted licence allows neither commercialisation nor modification of the original work.

## Results

Table S 2 lists the journals hosting the scientific production of ISS, IRE and INT, in the Q1, Q2, Q3 and Q4 ranges listed in the JCR Oncology category [6]. For each journal, the Table reports the publisher, business model and OA publication fee envisaged. The JRC’s subject category considered includes 182 journals, with an IF ranging from 94.333 (*Ca-a Cancer Journal for Clinicians*) to 0.101 (*UHOD-Uluslararasi Hematoloji-Onkoloji Dergisi*).

During 2010 the research staff of the three institutions published in 78 journals out of 182 with an IF ranging from 37.184 (*Nature reviews cancer*) to 0.364 (*Breast care*). Twenty-seven articles appeared in *Tumori,* a subscription-based journal and the official journal of INT, of which 24 were authored by INT researchers. The *Journal of experimental & clinical cancer research*, the official full OA journal of IRE, published 12 articles, 11 of them authored by IRE researchers.

Almost half (34) of the 78 journals were included in Q1, while 25 journals were found in Q2, 12 in Q3 and the remaining 7 in Q4. The large percentage of Q1 journals accounts for the high level of publications produced by the institutions concerned, in terms of prestige and impact of the chosen journals.

Of the total journals listed in Table S 2, the prevalent business models were the hybrid formula with a score of 51 journals, followed by 22 only subscription-based journals, and just 5 full OA journals. The last column shows that the highest IF is associated with a traditional subscription-based journal, while *Cancer cell*, ranked as the journal with the second highest IF, adopts a hybrid business model and charges the paid OA option of $ 5000 (€ 3864). Although, in some cases, the publication fees vary according to the type of article (i.e. original articles, reviews or letters), it is worth noting that, regardless of the quartile ranking, the most frequently charged fee is $ 3000 (€ 2318).

Table S 3 reports the copyright and self-archiving policies declared by publishers of the journals surveyed in Table S 2. As copyright rules established by the same publisher may include various models, Table S 3 also provides the links to the publisher copyright policy so that authors can access details of specific policies.

As expressly stated in their copyright policies, Table S 3 shows that half (12 out of 24) of publishers adopt a CTA; 4 out of 24 use a mixed system envisaging an ELF or a CTA, according to specific journals in their portfolios or to types of articles, and 4 out of 24 propose either a CTA or a CCA. In only one case (Nature Publishing Group) does the copyright policy provide an ELF or a CTA or a CCA according to the type of article (i.e. the CCA is used for articles reporting for the first time the primary sequence of an organism’s genome).

With reference to the range of colours reported by SHERPA/RoMEO database, Table S 3 shows that 6 out of 24 publishers are classified as “green”, 2 as “blue”, 8 as “yellow” and 5 as “white”. For three publishers no information was retrieved from SHERPA/RoMEO.

## Discussion

The remarkable number of Q1-ranked journals indicates the high level of publications produced by researchers and clinical staff of the three institutions involved in the study. This means that authors carefully consider IF values when deciding where to target their work, notwithstanding the widely-recognised biases of the raw IF value [12]. Research quality assessment is still a much-debated issue, also in the light of innovative parameters (i.e. webometrics [13]). This is not, however, the place to discuss this relevant topic and its impact on public health.

Where journal business models are concerned, it is worth mentioning that according to administrators of public funds and opinion leaders in the OA debate, the hybrid formula, which is based on a double income (subscription fees and article publication charges), is criticised for increasing publishers’ revenues while neither incurring any risk, nor reducing subscription costs. Publishers claim they will not “adjust” subscription costs until income from the paid OA option becomes steady. On the subject of publishing costs, Michael Jubb claims that “policymakers […] should also promote and facilitate a transition to gold open access, while seeking to ensure that the average level of charges for publication does not exceed circa £ 2,000” [14]. Notwithstanding the increasing number of publishers embracing the hybrid formula for their journals, it seems that economic revenues still remain low for publishers, compared with the income coming from subscription rates. According to Elsevier [15], the number of sponsored OA articles published in 2010 in its subscription-based journals, on payment of a publication charge of $ 3,000 per article, accounted for less than 1% (corresponding to 1114 articles). This low rate is probably due to the high cost of the sponsorship charge which, in some cases, is in addition to routinely charged author fees (costs of editing, colour charges, etc.). The paid OA option is thus not so affordable for authors, unless they can rely on funding from their own institutions or other public or private bodies. A remarkable number of articles authored by IRE researchers appeared in JECCR, a BioMed Central OA journal. This was probably due largely to the availability of funding provided by IRE in 2010 to institutional staff to cover their publication charges. This shows that decisions made at institutional level may have a strong impact on researchers’ publishing choices and, at the same time, represent a good opportunity to promote gold OA and wider visibility of institutional research findings.

With regard to OA publishing costs, it is interesting to note that, except in the case of the journal ranked second in Q1 (*Cancer cell),* which offers the highest paid OA option at $ 5000 (€ 3864), no relationship was found between IF ranking and article publication charges: in other words there was no correlation between more expensive fees and higher IF values. Thus, researchers should be aware that there are no additional economic costs to publishing in high-IF value journals compared with lower-IF journals. The publication fee most frequently charged by the journals surveyed for this article was $ 3000 (€ 2393) which is considerable when compared with the average publication fees ($ 900; € 718) for the journals listed in the multidisciplinary Directory of Open Access Journals (DOAJ) in 2010 [16]. The issue of cost-comparisons between OA journals and traditional subscription-based publications in times of financial constraint has recently been addressed by library administrators and other stakeholders [17]. Indeed, OA journals were initially welcomed as a “way of providing less costly alternatives to conventional journals” [17]. It was hoped that, in addition to allowing free access to the findings of science, the savings from cancelled subscriptions could exceed the publication fees charged by OA journals. However, this expectation of savings may be misguided, as the charges associated with the increased numbers of papers appearing in OA journals could lead to higher costs than in a traditional publishing environment.

The reasons and methods of meeting the financial costs of OA are still hotly debated. Once again, the recent Research Council UK (RCUK) and Wellcome Trust policies, which stress the need to ensure not just immediate access to but real reuse of published articles through CC-BY licensing, raise the issue of OA expenditure. In fact, their policies aim to promote the OA gold route by asking authors to cover the Article Processing Charges (APCs) while green OA supporters promote the lower cost of repositories in delivering access to research outputs. A crucial point of discussion is the transitional costs institutions currently have to meet for both subscriptions and publication charges. This means that, until now, investment in OA costs has not been compensated by a reduction in subscription costs for libraries. To address this problem, 6 the scientific community will have either to negotiate with publishers or to build consortia of institutions able to face the burden of costs. As pointed out by Neylon, “institutions need to take the opportunity to negotiate more imaginative and favourable arrangements with subscription publishers, to constrain transitional costs” [18].

Other rewarding ways to reduce publishing costs may be represented by free-software-based models such as the Open Journal System [19] (OJS), an open source journal management and publishing system, and by projects for national OAI-compliant repositories [20].

With regard to data relating to copyright rules, authors should be aware that the above models (CTA, ELF, CCA) may sometimes all be adopted by the same publisher, depending on different types of contribution (research articles, review articles, commissioned articles, etc.). Nature Publishing Group, for example, offers different kinds of licences, including the *Creative Commons Attribution non-commercial, Share alike* licence for articles reporting the primary sequence of an organism’s genome for the first time. Copyright rules adopted by the same publisher (either for OA or non-OA journals) may include various models. It is highly advisable to pay close attention to the information provided by each journal on copyright issues. This is particularly recommended for both OA and hybrid journals that require authors to pay a publication fee, as it is not always clear whether or not the author retains the entire copyright. When conditions for the re-use of contents are not clearly stated, uncertainty persists about which rights are actually granted “forcing users [the authors] to choose between the delay of seeking permission and the risk of proceeding without it” [21]. Given this situation, the standardisation of copyright licences would be welcome in order to provide a clear definition of the rights granted to authors of scholarly journals.

The data shown in Table S 3 refer to SHERPA/RoMEO colours of the surveyed publishers, revealing fewer (6 out of 24) publishers graded green and blue (most permissive conditions for self-archiving) compared with 13 out of 24 graded yellow and white (restrictive conditions or self-archiving not supported). It is worth noting that Elsevier and Springer are classified as green publishers, even allowing authors to deposit the published version (Pdf) of their journal articles in institutional repositories. This reveals a trend of major traditional publishers towards the OA business model, under pressure from the OA movement. However, this study shows that in the sample of the journals surveyed the yellow and white policies are still adopted by more than half of publishers, imposing restrictions on self-archiving practices.

The Directory of Open Access and Hybrid Journals [22] and the table provided by the Berkeley University Library, showing a selective list of OA and hybrid publishers [23], are two examples of tools (journal and publisher directories) for authors to enable them to identify at a glance the different OA models and detailed options offered by publishers. The latter represents a valuable effort by the library of an academic institution to support authors’ choices of suitable journals.

## Conclusions

The world of scientific communication has changed dramatically in the space of a few years. Print-based journals are now published electronically and their contents are immediately accessible without limits of time or space and without the burdensome expenses involved in the distribution of heavy paper-based publications. It has thus become more urgent, as well as necessary and possible, to disseminate research results rapidly and without the limitations in terms of costs and constraints associated with commercial rights. While awaiting future developments, researchers are enduring a period of transition in which it is no easy task to identify the best way to communicate their output.

Dissemination and access to research results continue to be of priority concern to leading scholars [24]. Before submission, a thorough evaluation of the factors listed in Table S 1 is highly recommended, given the wide variety of services delivered by publishers in “packaging” scientific literature to maximise visibility and usability. Each of the factors should be weighed in relation to subjective and contingent priorities affecting authors’ publishing practices (i.e. institutional targets and career-related considerations).

To date Italian authors have based their choices mainly on the IF of journals, in accordance with the approach to evaluating research adopted in the National Health System. Researchers are becoming increasingly aware that the impact of scientific work strongly depends on successful journal publication strategies. This is particularly important when considering the priorities of OA journals: to achieve rapid publication and the immediate dissemination of research results. It is no coincidence that many OA journals are gaining both visibility and higher Impact Factors.

Scientists have always sought to maximize the spread of their research results by publishing them in the most appropriate journals in the relative field. This means that they make the OA choice, accepting to pay APCs in pure OA or hybrid journals of good repute, in order to achieve faster circulation of their contents and immediate online access to their articles. In this regard, the issue of reusability of research outputs after publication is traditionally of less concern to authors compared with obtaining free access to “fresh” research literature. Notwithstanding this, major research funders such as the RCUK [25] and the Wellcome Trust [26] have recently stressed the point of “reuse rights” in their policies on open access, thus going beyond the concept of merely providing free access [18]. This implies that articles funded by these bodies and submitted for publication after 1 April 2013 in journals adopting an “author pays” model will be published under the Creative Commons Attribution Licence (CC-BY). In this way the priority route to reuse would seem to pass through the issue of licensing, which refer to the OA gold route (journals) rather than to the green OA channel (repositories).

With regard to authors’ self-archiving practices, they have proved to be effective when authors are “pushed” by a mandatory self-archiving policy, to archive their articles in an institutional or subject repository set up by the author’s affiliated institution or by a funding agency. Otherwise, when policies simply contain recommendations on a voluntary basis, authors are not sufficiently encouraged to post their articles, partly on account of restrictions still imposed by major scientific journals that do not allow the self-archiving of preprints, post prints or Pdf versions of published articles until an embargo period has expired.

Thanks to the principles supported by the worldwide OA movement, i.e. the removal of barriers to scientific publications, scientists and researchers are now called upon to play an active role in accelerating progress towards the goal of free science for all. Authors should be more aware of their rights to re-use their contributions, thus maximising the dissemination of published research results. To this end, they can show their commitment by submitting their papers to OA journals and by self-archiving them as e-prints in institutional or disciplinary repositories established by affiliated institutions. Both these forms of disseminating research findings represent consolidated methods to enhance the visibility and impact of scholarly literature. The OA publishing model is increasingly drawing authors’ attention to the high value of OA journals in which published papers are submitted to peer review in the same way that they are in non-OA journals. A still critical issue is that many OA journals require payment of a publication fee, thus making this model unsustainable for the individual researcher who is not supported by his institution or by research funds.

Within this framework, this articles addresses the need to acquire more knowledge concerning the strategies of OA journals. Researchers’ awareness of OA benefits is an ineluctable process, as highlighted by Stevan Harnad: “The freeing of their present and future refereed research from all access- and impact-barriers forever is now entirely in the hands of researchers” [27]. In line with this vision, this contribution intends to promote the synergy between researchers’ awareness of OA benefits and institutional policies mandating self-archiving practices.

## Competing interests

The authors declare that they have no competing interests.

## Authors’ contributions

EP and GC gave their contribution to the overall conception and design of the work and, together with EB, were responsible for drafting the article. EB and RS contributed to the analysis and interpretation of data, and contributed to the text revision and discussion. EP, TC, GC, RS and RR were responsible for the acquisition, checking and analysis of data displayed in the tables, while MF contributed in structuring and formatting data in the tables. All authors participated in the work for appropriate portions of the content and approved the final version of the manuscript.

## Supplementary Material

Additional file 1 Table S1 Key issues for author consideration when submitting a manuscript to a scientific journal. Click here for file

Additional file 2 Table S2 Journals hosting the scientific production of ISS, IRE and INT in 2010, ordered by IF quartile ranking (Q1-Q4). Note. 1) The currency in euros was calculated according to the exchange rate of 27 August 2012: 1 USD = €0.798028 €1 = 1.25309 USD checked at [http://www.xe.com/]. 2) Only "original research articles" are open access, while other types of articles appearing in the same journals are accessible on a subscription basis. Click here for file

Additional file 3 Table S3 Copyright policy of the publishers listed in Table S2. Click here for file
