# Supplementary material for: Where on earth to publish? A sample survey comparing traditional and open access publishing in the oncological field
Source: J Exp Clin Cancer Res. 2013 Jan 22;32(1):4. doi: 10.1186/1756-9966-32-4 (PMC3618298; doi:10.1186/1756-9966-32-4)
Supplement: Additional file 1 Table S1 — Key issues for author consideration when submitting a manuscript to a scientific journal. [file 1756-9966-32-4-S1.docx]

**Table S1:** Key issues for author consideration when submitting a manuscript to a scientific journal

| **Key issues** | **Sub-sections** |
| --- | --- |
| Journal topics and targeted audience | Journal subject category  Journal audience ( i.e. base, translational, clinical science, assistance, health policy, restricted niche) |
| Journal prestige | Impact factor within a given JCR subject category  Inclusion in major abstracting and indexing databases  Traceability by major search engines  Consolidated standards (i.e. broadly representative editorial board, critical refereeing system, high confidence level of scientists in the contents)  Technical quality of reprints and/or online display of articles  (i.e. layout, design, editing, print and binding)  Regular and trustworthy issuing |
| Costs related to publishing | Subscription costs  Costing for OA publication (paid OA option)  Manuscript submission fee  Additional charges for tables, color figures, extra pages  Relation between costs and other factors such as copyright policy, accessibility of articles, services offered |
| Copyright issues | Management of intellectual property rights  Self-archiving policy (permission granted to authors to post articles in public repositories, embargo period)  Negotiation of copyright conditions (i.e. SPARC addendum) |
| Publishing practice and process | Familiarity with a certain publisher or specific journal  Acquaintance with the editorial board of a journal  Clarity of instructions for authors  Friendliness of manuscript submission system  Traceability of manuscript submission process  Speed of publication process (time lapse between submission and publication) |
| Services offered by a journal | Language covered by a journal  Facilities provided by publishers to support authors (i.e. translating services)  Facilities in monitoring access (i.e. web usage statistics as download or online hit counts for articles)  Sharing of contents through social networks (Twitter, Facebook, Podcast etc.)  Access through mobile applications (i.e. smartphones or tablets)  Online user friendly access to the journal |
